# Reports on the Hospitals of the United Kingdom

**Published:** 1910-01-15

**Authors:** Henry Burdett


### REPORTS
### ON THE
### Hospitals of the United Kingdom.

By SIR HENRY BURDETT, K.C.B., K.C.V.O.

### XX.—CHELTENHAM GENERAL HOSPITAL.

This institution was established in 1839. Several of the wards exhibit the structural faults of that period. Attempts have been made to a moderate extent to improve matters here and there, but the institution bears evidence of too economical and narrow a spirit in matters of upkeep and appliances. It is due to the honour of so well-to-do a population as that of Cheltenham that its hospital should be modernised in directions we shall indicate later. It reflects great credit upon the resident staff in all departments that, despite the narrow spirit already referred to, the general appearance of the wards and offices should be as good as it is. On the whole they are well kept and administered by the staff, who ought to be encouraged by being given more up-to-date furniture and appliances in the wards and offices, and greater comfort and privacy in sleeping accommodation.

### Some Necessary Reforms.

The expenditure now proceeding on the reconstruction and enlargement of the surgical and medical wards in one wing has been wisely sanctioned. It will increase the number of beds to upwards of 100, and give the hospital some wards where light and air will be provided in fuller quantity than this hospital has known in the past. The enlarged wards have been decorated in wisely selected colours, and the design in green gives a charming effect. The Board should, however, take its courage in both hands and determine to refurnish these wards to a large extent, or at least to provide new lockers and glass tops for all the tables throughout the wards. A number of dressing trolleys, tin boxes for the surgical dressings, a modern test table or two, and other requisites now absent are essential to the equipment of each ward as a modern unit. The Board would be well advised, too, before the builders leave the premises, to enlarge the iron landing-stage of the fire staircase at the end of each ward and roof it in, so as to give abundant verandah space, to enable patients' beds to be wheeled out and placed there. Why this essential was not thought of and provided for in the original estimates, as it has been in every other hospital we have visited, passes our comprehension.

### The Chaplain's Fund.

We observe that the chaplain is apparently a permanent officer, and that the Board regrets the inadequacy of the subscriptions to the Chaplain's Fund. May this not be due to the general feeling which prevails nowadays that it is better for a hospital to make arrangements with the vicar of the parish in which the hospital stands for the religious services, rather than to maintain a special officer who cannot in the ordinary course have enough to do to occupy his whole time?

### The Nurses and their Quarters.

The Board in the last report states that it has from time to time reduced the number of nurses engaged on the private staff from 30 to 14. In our judgment this is not a step calculated to promote the best interests of the hospital. Sympathy is being destroyed by modern legislation to an increasing extent each year, and every voluntary institution is likely to find the result of the effect of this regrettable change in parliamentary methods expressed in a diminished and falling subscription list each year. To be safe financially, voluntary hospitals in the present day must meet the requirements of all classes of the population resident in the district which they serve. The supporters of every such hospital need at times the services of a trained nurse in their own homes, and the private nursing staff should be made available in the first instance for the benefit of such subscribers. The amount of annual subscriptions to a hospital each year tends under such a system to increase steadily. We hope, therefore, that the Board of the Cheltenham Hospital will speedily reverse its present policy in regard to the private nursing staff.

This hospital has one of the most inviting and most comfortably furnished relaxation rooms for nurses we have seen. Is this the only part of the nurses' accommodation which is usually shown to visitors? We imagine so, as the sleeping accommodation provided at this hospital for its nursing staff is a discredit to the board of management, for it is undoubtedly the very worst to be found anywhere in the South and West of England. Seeing, as we were informed, that most or all of the nurses are ladies by birth and education, we were amazed on visiting the so-called "Nurses' Home" to find it merely a small inconvenient building which contained nothing but cubicles. Not only were these cubicles small and objectionable in the sense that there could be little or no privacy for the occupants, but on the ground floor a portion of the cubicles had been recently shut off by a door and given up for the use of servants. Surely governors and even members of the Board must occasionally visit every part of the hospital. If this be so, many such visitors must have raised their voices and exerted their influence to secure for the nurses an adequate, up-to-date nurses' home where every nurse would be provided with a separate bedroom and ample bath and box accommodation. It is a grave reflection on the management to have permitted the present state of affairs to have arisen at all.

When we look back upon our inspection, and realise that without the exercise of great zeal and devotion on the part of the nursing and resident staff of this hospital the appearance and circumstances of the wards must have been mean, not to say contemptible, and that many comforts for the patients which a modern hospital ought to supply would have been wanting, it is a striking condemnation of the narrow spirit which seems to dominate the Board-room to find the accommodation here provided for the nurses the worst in the West, and probably about the worst in the whole of England. It is surely time that the governors took this matter in hand, that the medical staff on hygienic and humanitarian grounds should raise their voice in protest, and that the Board, either spontaneously or with the stimulus of continual prodding, should wake up and do its duty in this matter without a moment's avoidable delay.

**The Royal South Hants and Southampton Hospital** will be reported on next week.

